# Towards use of POCUS to evaluate hemodynamics in critically ill neonates: caution before adoption in this population

**DOI:** 10.1186/s13054-020-03394-4

**Published:** 2021-03-03

**Authors:** Patrick J. McNamara, Piers Barker, Amish Jain, Wyman W. Lai

**Affiliations:** 1 Division of Neonatology, University of Iowa, 200 Hawkins Drive, Iowa City, IA 52242 USA; 2 Duke University Medical Center, Durham, NC USA; 3 University of Toronto, Toronto, Canada; 4 University of California, IrvineIrvine, CA USA

**Letter to editor** regarding “International evidence-based guidelines on Point of Care Ultrasound (POCUS) for critically ill neonates and children issued by the POCUS Working Group of the European Society of Paediatric and Neonatal Intensive Care (ESPNIC)” by Singh et al. Critical Care (2020) 24:65

We read with interest the article by Singh et al. which outlined the role of point-of-care ultrasound (POCUS) in neonatal/pediatric intensive care units [1]. While we commend the authors for their efforts to better standardize indications for POCUS, we have three concerns: *First*, the presentation of recommendations for POCUS in neonates as evidence-based, rather than as author consensus; *second*, the inclusion of recommendations for critically ill neonates with older children; and *third*, the proposal that “these ESPNIC guidelines are developed for use by any neonatologist or paediatric intensivist.” Our intent is not to dissuade readers away from the use of POCUS but to restore balance between the desired recommendations and strength of available evidence and to offer additional suggestions.

(i) It is notable that only 2 recommendations achieve Quality of Evidence of A [(i)POCUS should not be used as a screen to diagnose congenital heart defects; (ii) assessment of ductus arteriosus patency], 4 reach level B (assessment of pulmonary artery pressures or pericardial fluid), while the remainder is classed as weak evidence. Surprisingly, the authors present strong agreement for all recommendations which creates an impression of greater validation than the evidence supports.
(ii) We believe the approach may inadvertently equate neonates to “small children.” Appraisal of cardiovascular physiology in critically ill neonates is challenging due to the complexity of the transitional circulation, unique interplay with neonatal disease and developmental variability of cardiovascular drugs. In addition, the reliability of subjective assessment of heart function or chamber size is questionable. We strongly believe that recommendations for POCUS in neonates be developed separately.
(iii) While availability of portable ultrasound machines has expanded, permitting the field to advance, related guidelines are incomplete. Published guidelines articulate the need for a well-defined training structure and guidelines for clinical practice [2–4]. Successful establishment of hemodynamic programs is attributed to the comprehensive nature of imaging protocols, exposure to higher case volume and organizational governance. The rates of attainment of imaging and interpretative competence are not congruent, and individual learning is also influenced by the complexity of pathophysiology and disease.
  A recent survey indicated that the establishment of hemodynamic programs is a high priority for neonatology leaders, as there is recognition that meticulous hemodynamic data are essential to optimize care [5]. At this juncture, leaders in POCUS and Neonatal Hemodynamics should strategize the scope of training and clinical application of cardiac POCUS to maximize the potential of this modality to improve patient care (Fig. 1).

**Fig. 1:**
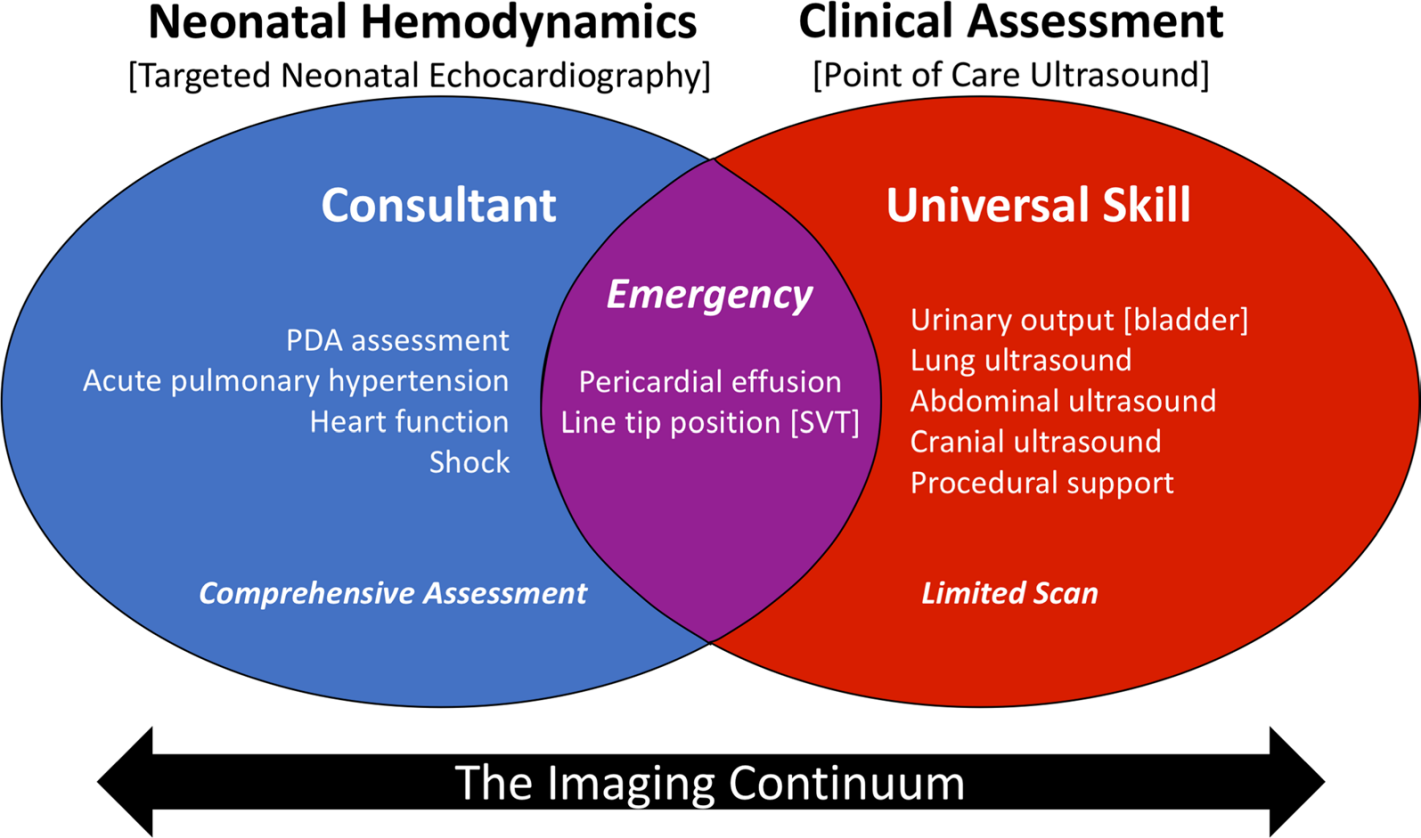
Proposed scope of integrated use of neonatal hemodynamics imaging and POCUS in the NICU

## Data Availability

Not applicable.
