# Supplementary material for: Towards use of POCUS to evaluate hemodynamics in critically ill neonates: caution before adoption in this population
Source: Crit Care. 2021 Mar 3;25:92. doi: 10.1186/s13054-020-03394-4 (PMC7927214; doi:10.1186/s13054-020-03394-4)
Supplement: Related texts

# Reply: Authors’ response

**Authors:** Y. Singh, C. Tissot, M. V. Fraga, N. Yousef, R. G. Cortes, J. Lopez, J. Sanchez-de-Toledo, J. Brierley, J. M. Colunga, D. Raffaj, E. Da Cruz, P. Durand, P. Kenderessy, H. J. Lang, A. Nishisaki, M. C. Kneyber, P. Tissieres, T. W. Conlon, D. De Luca

We thank the Editor for giving the opportunity to address the concerns and comments by McNamara et al.

*First*, we agree that a comprehensive hemodynamic evaluation involves echocardiographic assessment using many parameters including precise objective measurements [6]. Our published guidelines recommend the use of cardiac POCUS to assess individual components *within* a patient’s hemodynamic profile [7]. It refers to a basic, time-sensitive and focused echocardiographic assessment. For example, one of the indications includes “POCUS can be used for checking the patency of ductus arteriosus” which is only one consideration within the complex hemodynamic and physiologic evaluation of patent ductus arteriosus [8, 9]. Development of hemodynamic programs integrating subjective and objective echocardiographic measures within robust clinical experiences specific to the practice of neonatology is indeed a desired goal. Yet many neonatologists can answer fundamental, frequently asked, clinically relevant and lifesaving questions with basic POCUS training. Defining POCUS applications for the broader neonatal community and distinguishing applications for complete hemodynamic program development for neonatal specialists are important ongoing work, as we further discuss below.

*Second*, we would like to emphasize that these recommendations followed strict methodology consistent with other ESPNIC guidelines and are evidence-based, not expert-consensus guidelines [10]. We agree with McNamara et al. that some recommendations reached to a strong agreement despite “weak evidence grade.” Workgroups purposefully assessed recommendations beyond evidence within the literature since these data frequently “miss” the clinical context that can be provided by experts within a field. Many ultrasound indications have already become standard of care in daily clinical practice despite a prior absence of guidelines. For example, even in absence of high-grade evidence, any expert would agree that bedside ultrasound is the best and quickest imaging modality for the detection of pericardial effusion or to guide pericardiocentesis. Further, we can wait for the literature to demonstrate superiority of ultrasound-guided vascular access across all neonatal applications or accept that visualization of a needle tip as it enters a vessel is safer than non-visualization, and that the drive for “*quality of evidence A*” may ultimately impose unnecessary harm. This is similar to the use of echocardiography for diagnosing congenital heart defects (CHD) by a pediatric cardiologist; there is no disagreement that echocardiography is the investigation of choice to diagnose CHD despite the lack of published high-grade evidence.

*Third*, we agree with McNamara et al. that these guidelines are not for use by *any* neonatologist. Evidence-based guidelines have been prepared to define the clinical indications where POCUS can be used. The authors would agree that defining specific indications helps to prevent inadvertent use of POCUS in situations where its role is less clear or in settings where an adequate standard of care is lacking. For example, unlike neonatologist performed echocardiography (NPE) cardiac POCUS should not be used as a screening tool for congenital heart defects [7]. We believe that POCUS should be used *for specific clinical indications* and by *appropriately trained clinicians* [7]. Furthermore, we agree that paths translating training to competence at the bedside are necessary, and this work is ongoing within the POCUS community. Finally, POCUS is not an alternative to NPE or specialist echocardiography [7], which involves comprehensive training in hemodynamic evaluation and protocol-based assessments.

The purpose of guidelines is to guide the implementation of a tool within identified situations or settings where the care of a patient may be improved. The practice of neonatal critical care may be very different in different countries and regions, whether developing countries, American or European NICUs. We believe that with appropriate training, cardiac POCUS can be a useful tool for neonatal and pediatric intensivists to improve standards of care in critically ill children of all ages. Specific to Europe, a relevant proportion of units are mixed pediatric/neonatal critical care, and clinicians are trained to care for both types of patients.

There is no doubt that pathophysiologies amenable to POCUS applications bridge the neonatal-pediatric divide. We certainly agree that neonates are not small children, but are there more differences in physiology and physiopathology more than similarities? Many examples point to convergence rather than divergence of applications, demonstrated by the extension of adult lung ultrasound principles in the assessment of neonatal diseases.

We are in full agreement that cardiac POCUS, neonatologist performed targeted echocardiography and comprehensive echocardiography by the pediatric cardiologist are ‘the continuum’ of cardiac imaging. Compared to NPE, cardiac POCUS is designed for specific indications only, involves limited imaging and hence, relatively shorter training—allowing utilization of this important skill by more acute physicians in their clinical practice. The neonatal and pediatric intensivists performing both cardiac POCUS and NPE should work in close collaboration with the pediatric cardiologists as recommended in the guidelines (1–4). The consensus reached by the authors in developing our guidelines demonstrate the potential of this important collaboration, which we trust will enhance the care of critically ill newborns and children in the ICU environment. Working together they can share good practice and learn from each other while delivering the best possible care to their patients.
